# Supplementary material for: Curated and harmonised transcriptomics datasets of interstitial lung diseases
Source: Data Brief. 2025 Oct 14;63:112139. doi: 10.1016/j.dib.2025.112139 (PMC12581653; doi:10.1016/j.dib.2025.112139)
Supplement: Supplementary file 1 [file mmc1.zip › Supplementary_material/RNA-seq/fastQC reports/GSE184316/TRIMMED_SRR15926735_fastqc.html]

TRIMMED\_SRR15926735.fastq.gz FastQC Report 

FastQC Report

Sat 3 Dec 2022  
TRIMMED\_SRR15926735.fastq.gz

## Summary

- Basic Statistics
- Per base sequence quality
- Per sequence quality scores
- Per base sequence content
- Per sequence GC content
- Per base N content
- Sequence Length Distribution
- Sequence Duplication Levels
- Overrepresented sequences
- Adapter Content

## Basic Statistics

| Measure | Value |
| --- | --- |
| Filename | TRIMMED\_SRR15926735.fastq.gz |
| File type | Conventional base calls |
| Encoding | Sanger / Illumina 1.9 |
| Total Sequences | 8664958 |
| Sequences flagged as poor quality | 0 |
| Sequence length | 35-371 |
| %GC | 50 |

## Per base sequence quality

## Per sequence quality scores

## Per base sequence content

## Per sequence GC content

## Per base N content

## Sequence Length Distribution

## Sequence Duplication Levels

## Overrepresented sequences

| Sequence | Count | Percentage | Possible Source |
| --- | --- | --- | --- |
| AGGCTACACACTGAGGACTCTGTTCCTCCCCTTTCCGCCTAGGGGAAAGT | 72228 | 0.8335643404157297 | No Hit |
| CAATCCACTTGATCCCAACTCATCTCTCATTTATTTCGGCTTCTTTTATT | 67905 | 0.7836737350602276 | No Hit |
| CAGTGGGGCCACGAGCTGAGTGCGTCCTGTCACTCCACTCCCATGTCCCT | 63764 | 0.7358835438094449 | No Hit |
| GGGAAGCGGGGAATGTCTACGTGCGTATGCACGTGGCACTCTCTGCCCGA | 63303 | 0.730563264126612 | No Hit |
| CACCTCCCCGAAGCTCAGGGAGAGCCCTGTTAGGGCCGCCTCTGGCCCTA | 58451 | 0.6745676089832172 | No Hit |
| TTACAAAAGCAGAATAAAAGCGAAAAGAAATGAAAATGTTACACTACATT | 57805 | 0.6671122929851477 | No Hit |
| GGCATCAAAAGTGATGGACTGCCCATTGCTGGAGAAGACCTTCTCTCCTA | 56570 | 0.6528594829888384 | No Hit |
| AGACATCAAATCCTGCAGACAAGGGGAGCCCTCAGTCTGCAGGGCTCCAT | 49883 | 0.5756865757456643 | No Hit |
| TACCACTGCCACCTTCTCCATCGGCTCCACTGGCCTCGTGGTGTATGACT | 48804 | 0.5632341206962573 | No Hit |
| CTTCATGATGTAGCAGCAGGTGCCAGGGGCTGGCTTGTAGGCGATCAGCA | 43478 | 0.5017681562911211 | No Hit |
| TGATATGGTTCCTGGCAAGCCCATGTGTGTTGAGAGCTTCTCAGACTATC | 35238 | 0.40667248473679846 | No Hit |
| TGATGACACCCACCGCAACTGTCTGTCTCATATCACGAACAGCAAAGCGA | 34021 | 0.3926274080035933 | No Hit |
| CCCTGGCTGCTACAGGAGAATAGCAGACAGGTATAGTTAAGAAACAACTT | 20967 | 0.24197462930576236 | No Hit |
| AGCTGCTAAGTGCTGTGTTGTCGTTCCCCCTGCTTAAAATAAAGTTGTTT | 18874 | 0.21781986710149087 | No Hit |
| CAGCAGTTTACACCTGCTAGGGTGTTCAAAGGTCAGTGCTATAGAAATTC | 17087 | 0.19719657036998908 | No Hit |
| GGCAATGAGCGGTTCCGCTGCCCTGAGGCACTCTTCCAGCCTTCCTTCCT | 16573 | 0.19126463163468305 | No Hit |
| GTCCACGTCACACTTCATGATGGAGTTGAAGGTAGTTTCGTGGATGCCAC | 16267 | 0.1877331661619133 | No Hit |
| AACAAGCACAAAGCCAAGAAAACCAACGATGCCAGATACTGAATTTCTAT | 15592 | 0.17994316879550945 | No Hit |
| ACAAGAAGTACATCAAAGATTACATGAAATCAATCAAAGGGAAACTTGAA | 14930 | 0.1723032010080141 | No Hit |
| CTCTCTTTCTGGCCTGGAGGCTATCCAGCGTACTCCAAAGATTCAGGTTT | 14328 | 0.1653556774308658 | No Hit |
| GAAACCCAGACACATAGCAATTCAGGAAATTTGACTTTCCATTCTCTGCT | 13780 | 0.15903135364302978 | No Hit |
| ACGTTTACCTGTCCATGTCTTACTACTTTGACCGCGATGATGTGGCTTTG | 13681 | 0.15788882069595722 | No Hit |
| CAGCATGTTCCCTCTCCTCATGAGATTGGTGAAGAAAGTATTTGGCAAAG | 13111 | 0.15131060069766064 | No Hit |
| ATCAAAGAAGAACATGTGATCATCCAGGCCGAGTTCTATCTGAATCCTGA | 12210 | 0.14091239680561637 | No Hit |
| GTTCTGCAGCCCCTGTCATAAAAGGTTTTACTCTTTCTGGTCTCTGTTCT | 11895 | 0.13727706470129458 | No Hit |
| GCGTCTCTGTCTTCTTCAGTTTCGACTTATCGAATTTCTCGATCTCAGCC | 11660 | 0.13456499154410212 | No Hit |
| TCAGGAGACGCTCGTAGCCCTCGCGCTTCTCCTCGGCCAGTTCGCGGAAG | 11059 | 0.12762900870379293 | No Hit |
| TCAGGAGACGCTCGTAGCCCTCGCGCTTCTCCTCGGCCAATTCGCGGAAG | 10392 | 0.119931337232102 | No Hit |
| TGTGGATGAATCTTGAACTGAGTTCCACTTGTAAACTTCTTGTTTCTTGT | 10384 | 0.11983901133738904 | No Hit |
| CAATGGGGTACTTCAGGGTCAGGATGCCACGCTTGCTCTGGGCCTCGTCG | 9469 | 0.10927923712959715 | No Hit |
| TGCCATATCCACATGGAAAATCTCATCACCATCAAAGTCAAACATAAACT | 9037 | 0.1042936388150987 | No Hit |
| TCGCTCGTACTCGTGCGCCTCGCTTTGCTTTTCCTCCGCAACCATGTCTG | 8894 | 0.10264331344710499 | No Hit |
| GGAAGACAGTGTGGCGATTCCTCAGGGATCTAGAACTAGAAATACCATTG | 8893 | 0.10263177271026588 | No Hit |
| CATTGTGTTGTGCTAAACGCTGGGAGGGTACAAGTTGGTCATTCCTAAAT | 8849 | 0.10212398028934475 | No Hit |

## Adapter Content

Produced by FastQC (version 0.11.7)
